# The Use of Personal Protective Equipment: Finger Temperatures and Thermal Sensation of Workers’ Exposure to Cold Environment

**DOI:** 10.3390/ijerph15112583

**Published:** 2018-11-19

**Authors:** Adriana Seára Tirloni, Diogo Cunha dos Reis, Natália Fonseca Dias, Antônio Renato Pereira Moro

**Affiliations:** 1 Tecnological Center, Federal University of Santa Catarina, Florianópolis, Santa Catarina 88040-370, Brazil; diogo.biomecanica@gmail.com (D.C.d.R.); ergonomia.nd@gmail.com (N.F.D.); renato.moro@ufsc.br (A.R.P.M.); 2 Biomechanic’s Laboratory, CDS, Federal University of Santa Catarina, Florianópolis, Santa Catarina 88040-900, Brazil

**Keywords:** slaughterhouse, meat-packing industry, upper limb, personal protective equipment, discomfort, ergonomics

## Abstract

This study analyzed the finger temperatures of poultry slaughterhouse workers and its association with personal and organizational variables, bodily discomfort perception, and cold thermal sensation. The study included 143 poultry slaughterhouse workers. A thermograph was used to measure finger temperature and an interview to collect worker data. There were two groups: workers who used a tool and those without. The binary logistic regression, Friedman and Wilcoxon tests were used (*p* ≤ 0.05). Most workers presented at least one finger with an average temperature ≤15 °C (66.4%) and ≤24 °C (99.3%), perceived their cold hands, and wore three overlapping gloves (57.3%). There were no associations between finger temperatures (≤15 °C) and the analyzed variables. The chance of feeling cold for a worker who used a tool was greater than for a worker who did not (OR = 3.19, 95% CI 1.46; 6.94). There was a difference between the finger temperatures of each hand on both surfaces and the analyzed groups (*p* < 0.01). The temperature of each finger with its respective contralateral was different in the little fingers (no tool), index, middle, ring, and little fingers (using a tool) (*p* < 0.05). The use of several overlapping gloves was not sufficient to promote thermal insulation of the hands.

## 1. Introduction

Exposure to cold environments is a risk factor in industrial tasks [1], and in combination with other risks (repetition, force, awkward and static postures, and vibration), potentially increases development of work-related musculoskeletal disorders (WMSDs) [2]. According to the Brazilian Classification of Economic Activities (CNAE), the slaughtering of pigs, poultry, and other small animals is in 3rd place among the sectors that most develop occupational diseases in workers at the national level, leading this ranking in the southern states of Brazil [3].

Low temperatures are present in many types of indoor work, as in the food processing industry [4], and more specifically, in poultry slaughterhouses [2]. Cold is a hazard to health and may affect safety and performance of work [4]. Shortly after exposure to cold environments, a mediated vasoconstriction results in reduced blood flow to the peripheries in favor of a central blood pooling in the torso and deep body core [5]. Cooling, at a lower level, causes unpleasant sensations and thermal discomfort [6]. This discomfort may be a distracting factor, reducing the performance of tasks requiring concentration and vigilance, and may increase the risk of occupational accidents and injuries [6].

The skin temperature of the fingers tends to rapidly and exponentially decrease to a level approaching that of the ambient environment [7]. However, the cooling of the extremities (hands and feet) may increase while handling cold products or in contact with cold surfaces [4,6]. These conditions are routine in slaughterhouses, since European [8] and Brazilian standards [9] establish that the environment temperature of meat processing rooms does not exceed 12 °C, whereas processed meat cannot surpass 7 °C.

In these conditions, ISO 11079 Ergonomics of the thermal environment [10] institutes that finger temperatures ≤24 °C cause low physiological strain and ≤15 °C is considered high physiological strain. To detect thermal anomalies characterized by increasing or decreasing the temperature on the surface of the skin, some authors recommend the use of thermography because it is a non-invasive [11] and non-radiating tool [12]. Thermography has been used in studies related to occupational health, assisting in the diagnosis of musculoskeletal disorders [13], assessing the return to work [14], and analyzing the hand temperatures of office workers [15] and exposure to the cold environment [16,17].

The implementation of Brazilian Norm 36 (NR-36), concerning meat processing industries, aims to establish the minimum requirements for the evaluation, control, and monitoring of risks. This norm requires the adoption of individual and collective preventive measures—technical, organizational, and administrative—due to exposure to artificially cold environments, in order to provide thermal comfort to workers [18].

Several factors influence thermal comfort, such as air temperature and clothing insulation [19]. Occupational Safety and Health Administration (OSHA) [2] recommends the use and maintenance of effective Personal Protective Equipment (PPE), providing good-fitting thermal gloves, which can help with cold conditions while maintaining the ability to grasp items easily. Research has provided evidence that wearing gloves influences handgrip force, muscle activity requirement, and body posture [20,21].

Recent studies indicate that slaughterhouse workers presented low hand temperatures, despite wearing protective gloves [16,17], and bodily discomfort [22,23,24,25,26,27]. Considering that such studies are still scarce, there is a substantial knowledge gap regarding the finger temperatures of poultry slaughterhouse workers. Thus, the purpose of this study was to analyze the finger temperatures of the poultry slaughterhouse workers, and its association with personal and organizational variables, perception of bodily discomfort, and thermal sensation of cold.

## 2. Materials and Methods 

The Ethics Committee of the Federal University of Santa Catarina, Brazil (2098/11), in accordance with the Helsinki Declaration, approved this paper. It was conducted in a poultry slaughterhouse in the south of Brazil, with approximately 2500 workers. 

The participants worked in an artificially cold environment (8.8 °C to 11.5 °C—collected data by a company thermometer), handling products with temperatures between 0.9 °C and 11.3 °C (collected data by an infrared camera), and used PPE with a Certificate of Approval (CA) by the Brazilian Ministry of Labor (clothing, aprons, gloves, socks, and boots) provided by the slaughterhouse. According to the occupational safety and health staff of the slaughterhouse, the thermic gloves were changed on average every three months. Workers used five types of gloves during data collection: nitrile gloves, chain-mail gloves (stainless steel), cut protection gloves (polyester/synthetic), thermal-protection gloves (polyester), and polyethylene gloves. In this study, the tools the workers used were a knife with a knife sharpener (66.4%) and scissors (4.9%). All workers who used a tool wore a chain-mail glove on the nondominant hand (left) to protect from tool cuts. 

The daily working time was 7 h and 20 min, which included 60 min for a meal and four periods of 10 min for physiological necessities. 

### 2.1. Participants

The workers were randomly selected in the cutting sector of poultry meat, which included two work shifts. The requirement for participation in this research was that the worker would have to be performing the same activity at the workstation for at least 15 min prior to data collection, following the recommendations proposed by ISO 11079 [10]. In order to eliminate the possibility of skin temperature alterations, the following exclusion criteria were adopted: do not smoke [28], do not be sleep deprived prior to the assessment [29], no alcoholic beverages 12 h preceding data collection [30], and no females during menstrual period [31].

The investigation included 143 workers, 109 females and 34 males with a mean age of 32.3 years (18 to 55 years) and 28.9 years (18 to 54 years), respectively. 

### 2.2. Instruments

With the aim of recording the thermographic images (palm and dorsum of the hands), an infrared portable camera Flir^®^ T450SC (Flir Systems, Wilsonville, OR, USA) with an 18 mm lens was used. The Flir^®^ Tools software version 6.4.18039.1003 was used to analyze the images.

Questions regarding data to identify workers (age and gender), work organization (time working at the company, work shifts, glove use, and tool use), thermal sensation (perception of cold hands and felt cold in the hands), and upper-limb discomfort were also used.

Armstrong distinguishes between what is perceived and what is really felt [32]. He mentions that the cold is a sensation (perception), not something which exists apart from being sensed and which can correspond or not correspond to one’s impressions of it. It was adopted for this study that perceiving the cold hand would mean that the worker perceived the hands cold, but it did not mean that he/she felt cold in his/her hands, and vice versa, because it could “exist” or “not exist”.

Thermal comfort is defined by ASHRAE 55 [19] as that condition of mind that expresses satisfaction with the thermal environment and is assessed subjectively. Along these lines, thermal neutrality is cited as the indoor thermal index value corresponding with a mean vote of neutral on the thermal sensation scale. Vogt [33] points out that when the worker is subjected to cold stress, the effects (in seconds) are a cutaneous response (cooling) and discomfort. A numerical scale was used to evaluate the cold feeling in the hands, where zero indicated feeling neutral, and −1, −2, and −3 indicated feeling slightly cold to very cold [34].

### 2.3. Procedures

Data collection was performed at the workstations. The worker was instructed to stop his/her work, remove the gloves, and position his/her hands to capture the images.

**Figure 1:**
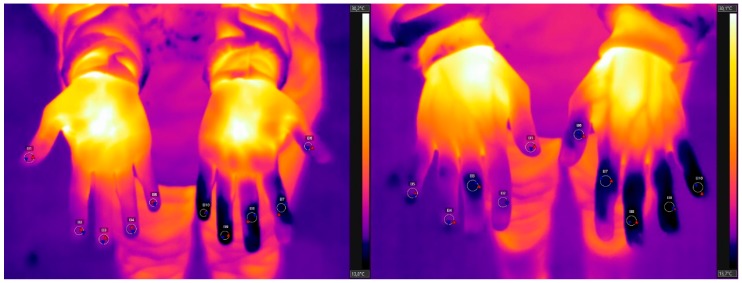
Thermographic images of the palm and dorsum, with the coldest areas of each finger identified.

The camera was positioned approximately 1.2 m away from the participant and 0.9 m above the floor. The temperature (8.8 °C to 11.5 °C) and humidity (50%) of the room were recorded for image analysis, and adopted an emissivity of 0.98 (human body). Two thermographic images of the palmar and dorsal surfaces of the hands were collected from each worker (Figure 1). 

The ellipse tool of the software was used to select the coldest area of each finger, with at least 7 pixels in diameter, avoiding the edges of the fingers. The mean temperatures of the fingers were extracted from the software for the subsequent analysis of the data. After capturing the thermographic images, each worker was asked about their personal identification data, work organization, thermal sensation of cold, and perception of bodily discomfort.

### 2.4. Data Analysis

The Shapiro–Wilk and Kolmogorov–Smirnov tests were performed to verify the normality of finger temperature data in two distinct groups of workers: those who used the tool (*n* = 102) and those who did not (*n* = 41). To compare the temperature values between the fingers of each hand, the paired Friedman test was applied. In order to compare the temperatures of each finger between the right and left hands, the Wilcoxon test was employed. 

The classification of the ISO 11079 “Ergonomics of the thermal environment” was used as a reference to determine the finger temperatures based on the physiological criteria described [10]. This norm states that finger temperatures ≤15 °C are considered high physiological strain, and ≤24 °C as low strain. The difference between the left and right hand was calculated considering the predetermined regions, and criteria adopted for acceptable limits of thermal asymmetry between both hands was <1 °C, as proposed by Hong et al. [35].

Binary logistic regression models were used to assess the association between the temperature of the fingers (≤15 °C or >15 °C) and the independent variables: age, gender, length of time working at the company, work shifts, number of gloves, tool use, perception of cold hands, felt cold in the hands, intensity of felt cold, and perception of bodily discomfort. They also verified the association between feeling cold and perception of cold hands, as well as with the use of tools.

Odds ratio and confidence intervals (95% CI) for temperature of the fingers were estimated for crude and adjusted analyses. First, the crude model was performed between the temperature of the fingers and the independent variables separately. In order for the variables to be included in the adjusted model, they should present *p* < 0.20 in the crude model. Based on the results of the crude analyses, the adjusted model included the following independent variables: age, work shifts, tool use, and perception of cold hands. 

All statistical tests were performed in IBM SPSS for Windows V.21.0 (IBM Corp., Armonk, NY, USA) and a *p* value ≤ 0.05 was considered significant for a comparison analysis.

## 3. Results

The majority of workers presented at least one finger with a mean temperature ≤15 °C (66.4%), regardless of their stratification in relation to the analyzed variables. Most workers were female (76.2%), used tools (71.3%), and perceived cold hands (72.7%), however, only 49% of workers felt cold in their hands at different intensities: mild (18.9%), moderate (21.0%), and very cold (9.1%). It was verified that in the two work shifts evaluated, the number of workers was equivalent, and 39.9% of the workers have worked in the company for one year or less and 32.1% for more than 10 years (1 month to 29 years). The workers wore one to five gloves on each hand, and the majority of workers wore three gloves overlapping (57.3%) (Table 1).

Bodily discomfort in the upper limbs was reported by 42.7% of workers, the shoulder being most frequently cited (30.8%), followed by the wrist (11.9%), hand (3.5%), and arm (2.8%). Only two workers complained of discomfort in their fingers (1.4%). 

Of the workers who had a finger temperature ≤15 °C, 77.9% perceived their cold hands. Regarding the workers who perceived their cold hands (*n* = 104), 60.6% perceived it in both hands and the minority perceived it only in the right hand (3.8%). Additionally, in relation to hand temperature classification, 32.9% of workers had finger temperatures between 15.1 °C and 24 °C and only one worker had finger temperatures >24 °C in both hands (0.7%).

There was an association between feeling cold in the hands (there was discomfort in relation to the cold) and perceiving cold hands (*p* < 0.001), in which the majority of the workers who perceived their cold hands felt cold in their hands (66.3%). The chance of a slaughterhouse worker who perceived that his/her hands were cold as well as felt the cold in his/her hands was 75 times greater than a worker who did not perceive his/her cold hands (OR = 74.91, 95% CI 9.87; 568.59). The chance of feeling cold for a worker who used a tool was 3.19 times greater than a worker who did not use a tool (OR = 3.19, 95% CI 1.46; 6.94).

Table 1 shows the results of the descriptive statistics (frequency and percent) and binary logistic regression. There were no associations between finger temperatures (≤15 °C) and personal and organizational variables, perception of cold, and bodily discomfort in workers in adjusted models.

It was observed that on the palm and dorsal surfaces of the hands in the group who did not use a tool, on average, 29% of the workers presented the lowest temperature in the little finger, being more frequent with respect to the other fingers. However, in the group who used a tool, on average, 40% of the workers had a lower temperature in the middle finger compared to the other fingers.

**Figure 2:**
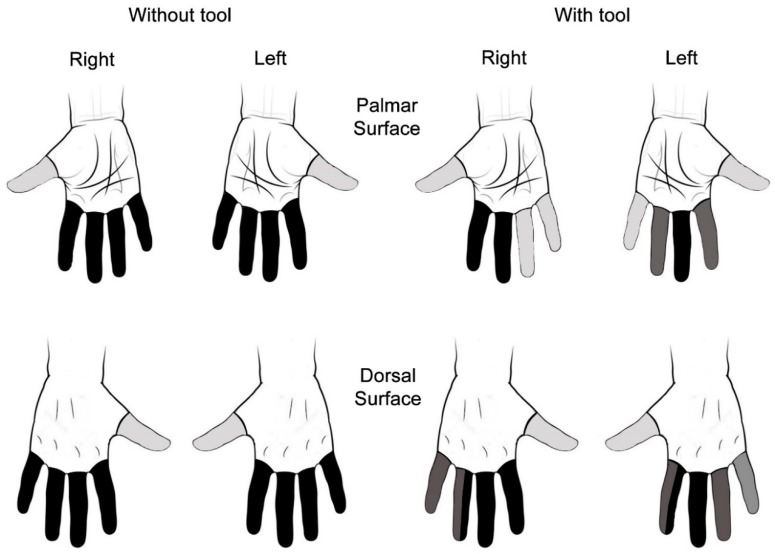
Difference between the finger temperatures in each hand, on the palmar and dorsal surfaces. Note: Fingers represented with distinct colors presented significant difference between the finger temperatures of each hand (*p* < 0.001).

The Friedman test was performed, which verified that there was a difference between the finger temperature on the palmar and dorsal surfaces in both hands in the two analyzed groups (*p* < 0.01) (Figure 2). In the group who did not use a tool, there was a difference between the thumb and other fingers on the same hand. Besides, in the group who used a tool, the middle finger was coldest on both surfaces and hands. When examining Figure 2, it is confirmed that in this group, the temperatures between the fingers were heterogeneous, since there were several ranges of temperatures (with tool—left hand on the dorsal surface—four colors). Overall, the thumbs presented higher temperatures than the other fingers (Figure 2).

The comparison between the temperatures of the hands (right and left) for the two groups is shown in Table 2. No significant difference was found between the temperatures of the fingers for most comparisons in the group who did not use the tool (*p* > 0.05). However, the little finger presented significantly lower temperature values for the left hands relative to the right in the palmar (*p* = 0.023) and dorsal surfaces (*p* = 0.018) (Table 2).

Regarding the workers who used the tool, there was no difference between the median values on the right and left sides only for the thumb on both hand surfaces. In the fingers that presented significant differences, the lower median values of the temperature were in the left hand (Table 2). As observed in Table 2, in both groups and hand surfaces, there were temperature differences (ΔT) > 1 °C between the right and left hands, in all fingers.

## 4. Discussion

In general, the majority of workers presented at least one finger with a mean temperature ≤15 °C (66.4%), in spite of whether or not workers used tools, with no association between these two variables. Therefore, most workers also had finger temperatures ≤24 °C (99.3%), which cause “low strain” and ≤15 °C is considered “high strain” [10]. The first condition is contraindicated for prolonged exposure, and the second, acceptable only in sporadic situations [4]. In the slaughterhouse analyzed, this recommendation was not fulfilled, it being understood that most workers were exposed to high thermal stress for prolonged periods. Other parameters are presented by Vogt [33], in which the temperature of hands and fingers between 15 and 20 °C causes a decrease in the simple work performance of these regions and an occasional painful sensation. With the temperature between 10 and 15 °C, there is a gross muscular force decrease, muscular coordination deterioration, and painful sensation. The author also mentions that for good functioning of the hands and fingers, their temperatures must be between 32 and 36 °C, which is a more conservative parameter than that adopted by ISO 11079 [10]. 

Research was conducted in two fish processing companies with 52 workers, where the air temperature at the head level of the workers was 22–24 °C, while at the feet level was 10–13 °C. The fish meat temperature handled by the workers was 6–7 °C, and the finger temperature of the fillet cutters was 9–11 °C, even using protective gloves [36]. It was observed that in the present paper, the ambient temperature was close to the temperature at the feet level in the fish slaughterhouse (8.8–11.5 °C) and presented a higher range for the product temperature (0.9–11.3 °C), as well as the finger temperatures (8.8–30.3 °C). Regardless of the differences, it was found that in both studies there was an inefficiency of the PPE concerning the adequate thermal insulation of the hands.

The results of the present study corroborated with other studies on the subject of temperature differences between the respective contralateral fingers [16,17]. It was verified that most workers who used a knife presented temperature differences greater than 1 °C between hands (right and left) for the fingers on both hand surfaces, unlike the group that did not use a knife.

Studies agree with our findings regarding the group that did not use a tool [17,37], where the little finger had lower temperatures between the fingers of each hand on both surfaces, and the thumb was the hottest finger in all conditions analyzed. 

In healthy adults, Gatt et al. [37] revealed patterns in temperature variation across fingers; when considering the hands, a monotonic decreased temperature from the thumb to the little finger may be noted. The examination of the temperature of each finger in pig slaughterhouse workers showed that the highest frequency of finger temperatures ≤15 °C was in the right little finger (10%) on the dorsal surface in the group who did not use a knife [17]. Conversely, for the workers who used a knife, the finger temperature frequency ≤15 °C was higher in the hand that handled the products (left) on the dorsal surface for middle and ring fingers (19% for each) [17]. This result is similar to this research, since the middle finger presented lower temperatures between the fingers of each hand for this worker group.

When comparing the finger temperatures between the contralateral hands, it was verified that the workers who did not use a knife lacked a significant difference between the right and left hands, except in the little finger. There were no significant differences between the proportion of workers who had hand temperatures ≤24 °C and >24 °C (right and left) in the worker group who did not use a knife, both in the poultry slaughterhouse [16] and in the pig slaughterhouse studies [17]. Gatt et al. [37] analyzed 63 healthy adult participants and confirmed that for finger and toe extremities of the volar surface, the mean temperatures of the left and right sides of participants were considerably similar in terms of both magnitude and pattern.

In contrast, workers using a knife showed temperature differences in all the fingers except the thumb in this paper. Substantially lower median values for the left hand were found on both surfaces. In a study with pig slaughterhouse workers, there was a significant temperature difference in all fingers as well as ulnar and radial contralateral regions [17]. 

An analysis with different results evidenced that in the poultry slaughterhouse, there were significant differences for the thumb, index, middle, and ring fingers on the palmar and dorsal surfaces in those who used a knife [16]. For the pig slaughterhouse workers, the difference occurred in the middle (*p* = 0.048) and little fingers (*p* = 0.026) on the dorsal surface, and in the index (*p* = 0.028) and little fingers (*p* = 0.021) on the palmar surface [17]. In both studies, the non-dominant hand (hand that handled the cold meat) presented a higher percentage of workers with finger temperatures ≤24 °C than the respective fingers of the hand that held the knife, corroborating these findings.

Conforming to Ramos et al. [16] and Tirloni et al. [17], the non-difference between the finger temperatures (right and left) in the group that did not use a tool is related to the characteristics of the activity that require direct contact and/or handling of cold products with both hands. Cooling of the extremities (head, hands, and feet) may increase during the handling of cold products or in contact with cold surfaces [4,6].

In the case of the researched slaughterhouse, besides the workers using a tool to handle (touch) the product with only one hand (left), they also used a chain-mail glove on this hand, a factor that may have aided in the increased cooling of the fingers of that hand. The use of a chain-mail glove (cut resistant) on their nonknife hand by meat processing industry workers was perceived as uncomfortable and resulted in the cooling of the hand when used in a cold environment [38].

Mild or intense physical exposure to cold depends on the balance between internal heat production (as a result of physical work) and heat loss, protective clothing, and climatic conditions that determine the amount of heat lost [33]. As reported by Geng et al. [39], metals are highly conductive surfaces. In this regard, the energy transfer rate from the body (hand) to the metal (chain-mail glove) can occur quickly if the gloves used under it do not provide adequate thermal insulation.

In Brazil, for the hand protection against sharp and piercing agents, chain-mail gloves or gloves made of other materials should be used [40], based on the application of ISO 13999-1 [41] and ISO 13999-2 [42]. Chain-mail gloves are used in those aspects of work where a knife is moved towards the user’s hand and forearm, especially when working with hand knives in slaughterhouses, in large-scale catering establishments, and in manual boning-out operations to process meat and poultry [41]. In work where the knife is generally used to cut away from the hand, or the knives are not finely pointed, it may be appropriate for ergonomic reasons to use gloves that are more comfortable by providing less protection than that furnished by products fulfilling the requirements of ISO 13999-1 [42]. 

The chain-mail glove, whilst seen to add protection from knife cuts, was generally disliked by meat workers due to: discomfort and poor fitting to the range of male/female hand sizes; inadequately fitting finger tips resulting in lack of grip sensitivity for the fingers; the chain-mail getting cold and chilling the hand in 10 °C temperature-controlled work rooms; weight of glove; fatiguing of hand/arm, particularly when worn with full chain-mail forearm guard; and no availability of left-handed model [38].

Most of the workers used three overlapped gloves, however, the majority of the fingers had temperatures ≤15 °C. This may have occurred because of the ineffectiveness of the thermal insulation of the gloves used (glove quality), although they had a Certificate of Approval issued by the Ministry of Labor, and/or by the use of a chain-mail glove by workers (on non-dominant hand) and/or by the order of the worn gloves when overlapped.

NR-36 states that the employer must provide work clothes so that workers can have more than one piece to use in an overlapping manner, at their discretion, and according to the activity and workplace temperature, taking into account the hygienic characteristics and thermal comfort [18].

The gloves used in slaughterhouses must be compatible with the nature of the tasks, the environmental conditions, and the hand size of the workers, besides being substituted, when necessary, in order to avoid compromising their effectiveness [18]. According to the work safety staff of the analyzed slaughterhouse, the thermic gloves were changed on average every three months.

Studies have also found that workers used three overlapping gloves on the left (56.6%) and right (60.5%) hands [27] and each one with different functions [38]. Willms et al. [21] mention that to maintain an unloaded grip posture and to create a fixed submaximal force, participants increased muscle activation for all muscles with increasing glove thickness (*p* < 0.05). The poultry slaughterhouse workers perceived the exertion applied when cutting the product as mild (48.7%) and moderate (42.1%); there was no correlation between the number of overlapped gloves on the same hand and the intensity perception of applied force (*p* > 0.05) [27].

In a study conducted with 150 workers from five slaughterhouses in Australia, it was suggested that one water-resistant glove under the chain-mail glove on the non-knife hand could be required in order to help keep the hand dry and warm from the cold-conductive chain-mail glove [38].

The NR-36 manual [43] recommends that in work activities where clothing may become wet, the outside is impermeable and the layer closest to the body is insulated and removes moisture from the skin in order to maintain dryness. Risikko et al. [36] cite that replacement of the inner cotton glove worn inside the latex gloves with a polypropylene one also reduced the problems of painfully cold fingers for the filet cutters in fish slaughterhouses.

In meat processing, meat cutting, and boning in a workplace with low temperatures (−10 °C to 6 °C) that involves small objects (meat pieces) with frozen or wet surfaces, the glove materials should have hydrophobic properties characterized by considerable wetting resistance (degree 4 or 3), good thermal resistance (class 3 or 2), and favorable ergonomic properties (low bending modulus) due to the high degree of manual dexterity required in this type of work [44].

Human adaptation to cold may occur through acclimatization or acclimation and includes genetic, physiologic, morphological, or behavioral responses that can be either inherited or acquired and both types can result in morphological and/or physiological changes [45]. In the present research, the subjective evaluation (perceiving cold hands) had no association with a temperature ≤15 °C, but had to feel cold in the hands. This means that this subjective assessment of slaughterhouse workers could be used to evidence some discomfort in relation to the cold, since most of the workers who perceived the cold hand felt cold in their hands.

Fish slaughterhouse workers presented low finger and foot temperatures (9–11 °C), and it was confirmed both by the workers’ subjective evaluations (questionnaire) as well as the temperature measurements performed. The most striking finding was that 96% reported some level of discomfort (feeling cold) for the fingers and 88% for the feet. Periodical circulatory disturbances in hands and/or feet were reported by 52% of the respondents [36].

Most humans experience a thermal neutrality sense when exercising very quick or sedentary work at an ambient temperature of 20–26 °C with proper clothing [33]. It is considered an artificially cold environment in slaughterhouses when the temperature is lower than 10–15 °C, depending on the region of Brazil where the plant is located [18]. Studies in slaughterhouses found that the workers presented some discomfort in relation to the cold, in general and in the hands, when exposed to artificially cold environments [17,23,25,26,27].

A study with 290 poultry slaughterhouse workers established that 41.4% felt cold [23], and in another research with 72 pig slaughterhouse workers, cold was felt by 44.4% [26]. In opposition, an appraisal of 312 poultry slaughterhouse workers corroborated that the majority felt cold during the workday (62.5%) [25].

In a cold environment, the thermal sensations for the body parts vary widely, wherein the hand and foot are rated as significantly colder than the head (paired *t*-tests, *p* < 0.001) and trunk (back, chest, and pelvis, *p* < 0.03), followed by the arms (*p* < 0.05) [46].

The results of the present paper showed that almost half of the workers felt cold in the hands (49%), corroborating with other studies [23,25,26]. The body regions that most workers complained of feeling cold were hands (35%) and feet (31.7%) [23]. For pig slaughterhouse workers, it was the feet (68.8%) and the hands (28.1%) [26], and in another research, it was again the feet (56.2%) and the hands (42.8%) [25]. In all previous studies, the hands presented less than half of the cases, however, in a poultry slaughterhouse (*n* = 76), 61% of the workers felt cold in their hands [27], 78% of the poultry slaughterhouse workers felt cold at different intensities: mild (18.1%), moderate (29.5%), and very cold (30.4%) [16]. One study with 106 pig slaughterhouse workers verified that 66% felt cold in the hands with intensities: mild (25.5%), moderate (16.0%), and very cold (24.5%) [17]. In this analysis, although most workers had hands ≤15 °C, only 9% of workers felt very cold in the hands.

In the present research, there was an association between the tool use and cold feeling in the hands, and those who used a tool at the slaughterhouse had a greater chance of feeling cold in the hands. Tirloni et al. [26] stated that the group who did not use the knife had significant positive correlations between the thermal sensation of the hands (intensity of feeling cold) and the temperatures of the fingers on both hands. However, for workers who used the knife, these correlations were noted only in the fingers of the left hand, on both surfaces. Additionally, there was an association between the use of a knife and the thermal sensation reported by the pig slaughterhouse workers (*p* = 0.001), as the majority of these workers who used the knife felt cold in their hands (81%). In opposition, Ramos et al. [16] obtained significant positive correlations between the thermal sensation of the hands and the finger temperatures found only in the group of workers who did not use the knife; however, there was no significant association between the tool use and feeling cold in the hands.

Working in a cold environment is a risk factor for developing WMSDs [2], and body discomfort was associated with the perception of cold, being *p* = 0.035 [23] and *p* < 0.001 [25]. In two frozen food factories, factors affecting episodic finger symptoms (darkening and reddening of fingers, finger and toe pain) were sex, duration of work, and work section. Female workers had more abnormal symptoms than males (OR = 1.645, 95% CI: 1.119–2.419) and warehouse workers had more abnormal symptoms than office workers (OR = 13.514, 95% CI: 5.169–35.327), where the temperatures were normally the lowest at −18 °C and equal to 25 °C, respectively [47].

Studies confirm that poultry slaughterhouse workers felt bodily discomfort, which showed that 67.2% felt discomfort in at least one body region; the shoulder was the most frequently cited (62.6%) and the hand was the least (25.6%) [23]. In another poultry slaughterhouse, 43% of the respondents had discomfort symptoms, mainly in the shoulder (29%), and the hand with 22% [24]. In addition, among the 312 workers interviewed, 71.2% reported bodily discomfort, 50.3% in the shoulder and 21.8% in the hand [25]. Evaluating 76 workers, Tirloni et al. [27] identified that 54% of the workers felt discomfort in their upper limbs, 38.8% in the shoulder, and 28.9% in the hand. A study conducted with 90 poultry slaughterhouse workers identified that for regions with discomfort, the shoulder was most mentioned (45%), and the wrist and hand had 20% of the cases [22]. The same happened with pig slaughterhouse workers; 83.3% felt bodily discomfort and the regions most affected were shoulders (47.2%) and the hand was the least (18.1%) [26]. Therefore, in only one investigation [24], most workers did not feel bodily discomfort, however, in all studies, the shoulder was the most referenced region and the hand was cited by 18–29% of the workers.

Some studies, including this one, found that the left hand presented lower temperature values in relation to the right in slaughterhouse workers who used a tool [16,17]. Other studies showed that these workers felt most discomfort in the right side of the body (*p* < 0.05) [25] in most workers (73.6%) [25], and presented a higher risk of developing WMSD in the right upper limb [48,49].

Although there were no associations between the finger temperatures and the analyzed variables, the other results of this study serve as an alert to the low temperatures to which the slaughterhouse workers are exposed (cold environment, chain-mail glove, and handling of cold products), which require preventive measures regarding the adequate thermal insulation of the hands. One of these measures may be established as a standard procedure for monitoring glove conditions, replacing them where necessary.

## 5. Conclusions

Most workers presented at least one finger with an average temperature ≤15 °C, perceived their cold hands, and wore three gloves on each hand. There were no associations between finger temperatures and the analyzed variables.

Slaughterhouse workers who perceived their cold hands had more of a chance to feel cold in the hands than those who did not perceive their cold hands. Likewise, the chance of feeling cold for a worker who used a tool was greater than for a worker who did not use a tool.

The most frequent fingers that presented lower temperatures in each hand were the little finger in the group that did not use a tool and the middle finger in those that used a tool, for both hand surfaces. In the group who did not use a tool, the thumb was hotter than the other fingers of the same hand. Moreover, in the group who used a tool, the middle finger was colder than the other fingers of the same hand, on both surfaces and hands.

On the palm and dorsal surfaces, only the little finger presented a difference between the hands (right and left) for the workers who did not use a tool. However, in the group who used a tool, there was a difference between the index, middle, ring, and little finger temperatures and the respective contralateral fingers. Overall, the lower median values of the temperature were in the left hand.

The results showed that, despite the workers using several overlapping gloves on each hand, the protection of this bodily region was not achieved concerning the low temperatures of the environment and the manipulated products, as there was not adequate thermal insulation of the hands. Therefore, it is suggested that the Brazilian institutions responsible for approving personal protective equipment should review glove models indicated for use in slaughter and meat processing industries. Additionally, it is recommended that manufacturers of these types of gloves invest in the development of materials with greater thermal insulation and good fitting, maintaining the ability to grasp and reduce the effects of this risk factor on the health of slaughterhouse workers.

## Figures and Tables

**Table 1:** Frequency and percent of the variables in relation to hand temperature and crude and adjusted associations between finger temperature and personal and organizational variables, as well as thermal sensation of cold and bodily discomfort.

Variables	Finger Temperature (≤15 °C)			Crude		Adjusted ^#^	
	Yes	No	Total	OR	*p*-Value	OR	*p*-Value
	*n* (%)	*n* (%)	*n* (%)	95% (CI)		95% (CI)	
**Age** (mean ± SD)	30.6 ± 10.6	33.3 ± 10.2	31.5 ± 10.5	0.98 (0.94; 1.01)	0.143 *	0.97 (0.94; 1.00)	0.073
**Gender**					0.284		-
Female	75 (68.8)	34 (31.2)	109 (76.2)	1.54 (0.70; 3.42)			
Male	20 (58.8)	14 (41.2)	34 (23.8)	Ref.			
**Length of time working at the company** (mean ± SD—years)					0.885		-
	4.0 ± 5.9	4.0 ± 5.7	4.0 ± 5.9	1.00 (0.95; 1.07)			
**Work shifts**					0.030 *		0.061
First	41 (57.7)	30 (42.3)	71 (49.7)	0.46 (0.22; 0.93)		0.48 (0.22; 1.03)	
Second	54 (75.0)	18 (25.0)	72 (50.3)	Ref.		Ref.	
**Number of gloves** (mean ± SD)					0.484		
	3.2 ± 0.6	3.1 ± 0.8	3.1 ± 0.7	1.20 (0.72; 2.00)			-
**Tool use**					0.099 *		0.408
No	23 (56.1)	18 (43.9)	41 (28.7)	0.53 (0.25; 1.13)		0.71 (0.31; 1.61)	
Yes	72 (70.6)	30 (29.4)	102 (71.3)	Ref.			
**Perception of cold hands**					0.053 *		0.111
No	21 (53.8)	18 (46.2)	39 (27.3)	0.47 (0.22; 1.01)		1.90 (0.86; 4.19)	
Yes	74 (71.2)	30 (28.8)	104 (72.7)	Ref.		Ref.	
**Felt cold in the hands**					0.377		-
No	46 (63.0)	27 (37.0)	73 (51.0)	0.73 (0.36; 1.47)			
Yes	49 (70.0)	21 (30.0)	70 (49.0)	Ref.			
**Felt cold in the hands (intensity)**					0.618		-
No	46 (63.0)	27 (37.0)	73 (51.0)	0.51 (0.13; 2.02)			
Mild	17 (63.0)	10 (37.0)	27 (18.9)	0.51 (0.11; 2.31)			
Moderate	22 (73.3)	8 (26.7)	30 (21.0)	0.83 (0.18; 3.78)			
Very cold	10 (76.9)	3 (23.1)	13 (9.1)	Ref.			
**Bodily discomfort**					0.597		-
No	53 (64.6)	29 (35.4)	82 (57.3)	0.83 (0.41; 1.68)			
Yes	42 (68.9)	19 (31.1)	61 (42.7)	Ref.			
Total	95 (66.4)	48 (33.6)	143 (100.0)				

^#^ Adjusted for age, length of time working at the company, work shifts, tool use, and perception of cold hands. * Factors were removed from the logistic model (*p* > 0.20); Ref.—Reference.

**Table 2:** Comparison between finger temperatures (right and left) and the use of a tool by workers.

	Without Tool								
	Minimum		Maximum		Median			ΔT	
Palmar Surface	R	L	R	L	R	L	p	Mean	SD
Thumb	13.0	13.5	27.8	26.8	17.6	17.2	0.301	1.3	1.5
Index	11.9	12.1	25.5	27.6	16.0	15.6	0.717	1.5	1.3
Middle	12.0	11.8	25.5	27.0	15.9	15.4	0.645	1.5	1.5
Ring	11.8	12.2	27.5	27.4	15.8	15.3	0.123	1.5	1.4
Little	11.2	10.7	28.8	26.9	15.7	15.4	0.023 *	1.9	2.5
**Dorsal Surface**									
Thumb	12.1	12.3	29.6	28.6	18.6	18.1	0.759	1.3	1.3
Index	12.8	12.3	27.4	27.7	16.1	16.4	0.615	1.4	1.3
Middle	12.3	12.4	27.0	27.6	16.5	16.4	0.309	1.5	1.4
Ring	12.7	12.2	27.3	26.7	16.6	16.2	0.685	1.2	1.6
Little	12.1	11.8	27.7	26.7	16.3	15.5	0.018 *	1.8	2.5
	**With tool**								
	**Minimum**		**Maximum**		**Median**			**ΔT**	
**Palmar Surface**	**R**	**L**	**R**	**L**	**R**	**L**	**p**	**Mean**	**SD**
Thumb	12.7	13.2	28.8	30.3	17.7	17.4	0.783	2.0	1.5
Index	12.2	12.3	25.6	26.3	16.5	16.0	0.042 *	2.0	1.3
Middle	12.9	13.0	27.1	27.0	16.5	15.7	<0.001 *	2.2	1.5
Ring	13.3	12.8	27.6	26.5	16.8	16.1	<0.001 *	2.6	1.4
Little	14	13.9	29	26.9	17.2	16.8	<0.001 *	2.6	2.5
**Dorsal Surface**									
Thumb	11.1	10.9	28.5	26.1	16.7	16.9	0.312	1.9	1.3
Index	9.6	9.6	23.8	23.7	15.5	14.9	0.001 *	2.2	1.3
Middle	8.8	9.0	24.5	24	15.5	14.7	<0.001 *	2.1	1.4
Ring	9.3	9.7	28.9	24.8	15.7	15.0	<0.001 *	2.3	1.6
Little	10.2	10.6	29.3	27.6	15.6	15.2	0.001 *	2.3	2.5

R—right; L—left; * *p* ≤ 0.05; ΔT—Difference between temperature averages of the fingers (right and left).
